# *Spilanthes filicaulis* (Schumach. & Thonn.) C. D Adam leaf extract prevents assault of streptozotocin on liver cells via inhibition of oxidative stress and activation of the NrF2/Keap1, PPARγ, and PTP1B signaling pathways

**DOI:** 10.1371/journal.pone.0306039

**Published:** 2024-06-26

**Authors:** Oluwafemi Adeleke Ojo, Fiyinfoluwa Stephen Oladepo, Akingbolabo Daniel Ogunlakin, Damilare IyinKristi Ayokunle, Adeshina Isaiah Odugbemi, Dare Ezekiel Babatunde, Adebola Busola Ojo, Omolola Adenike Ajayi-Odoko, Basiru Olaitan Ajiboye, Samuel Olatunde Dahunsi

**Affiliations:** 1 Phytomedicine, Molecular Toxicology and Computational Biochemistry Research Laboratory (PMTCB-RL), Biochemistry Programme, Bowen University, Iwo, Nigeria; 2 Pure and Applied Biology Programme, Bowen University, Iwo, Nigeria; 3 Anatomy Programme, Bowen University, Iwo, Nigeria; 4 Department of Biochemistry, Ekiti State University, Ado-Ekiti, Nigeria; 5 Microbiology Programme, Bowen University, Iwo, Nigeria; 6 Department of Biochemistry, Federal University, Oye-Ekiti, Oye-Ekiti, Nigeria; National Institutes of Health, UNITED STATES

## Abstract

**Background:**

*Spilanthes filicaulis* (Schumach. & Thonn.) C. D Adam is a shrubby plant of the Asteraceae family that has medicinal benefits for the pharmaceutical and cosmetic industries.

**Purpose:**

The purpose of this study was to assess the effectiveness of *Spilanthes filicaulis* leaf extract in a streptozotocin (STZ)-induced rat model and the associated signaling pathways.

**Methods:**

A sample of 25 male Wistar rats was randomly assigned to groups I, II, III, IV, and V. Each group included five animals, i.e., control rats, diabetic control rats, diabetic rats treated with metformin, and diabetic rats treated with 150 mg/kg/bw and 300 mg/kg/bw of the methanolic extract of *S*. *filicaulis* leaves (MESFL). Treatment was administered for 15 successive days via oral gavage. After 15 days, the rats were evaluated for fasting blood glucose (FBG), glycated hemoglobin (HbA1c), alanine aminotransferase (ALT), aspartate aminotransferase (AST), alkaline phosphatase (ALP), triglyceride (TG), total cholesterol (TC), low-density lipoprotein (LDL), high-density lipoprotein (HDL), reduced glutathione (GSH), glutathione-S-transferase (GST), superoxide dismutase (SOD), catalase (CAT), lipid peroxidation (MDA), hexokinase, and glucose-6-phosphatase activities. Gene expression levels of nuclear factor erythroid 2-related factor 2 (Nrf2), peroxisome proliferator-activated receptor gamma (PPAR-γ), kelch-like ECH-associated protein 1 (Keap1), protein tyrosine phosphatase 1B (PTP1B) and the antiapoptotic protein caspase-3 were examined.

**Results:**

MESFL was administered to diabetic rats, and changes in body weight, fasting blood glucose (FBG) and HbA1c were restored. Furthermore, in diabetic rats, *S*. *filicaulis* significantly reduced the levels of triglycerides (TGs), total cholesterol (TC), low-density lipoprotein (LDL), and very low-density lipoprotein (VLDL) and significantly increased HDL. *S*. *filicaulis* improved ALT, AST, and ALP enzyme activity in diabetic rats. MDA levels decreased considerably with increasing activity of antioxidant enzymes, such as GST, SOD, CAT and GSH, in diabetic liver rats treated with *S*. *filicaulis*. Diabetic rats treated with MESFL and metformin exhibited upregulated mRNA expression levels of nuclear factor erythroid 2-related factor 2 (Nrf2) and peroxisome proliferator-activated receptor gamma (PPAR-γ). Kelch-like ECH-associated protein 1 (Keap1) and protein tyrosine phosphatase 1B (PTP1B) mRNA expression in the liver was downregulated in diabetic rats treated with MESFL and metformin. In addition, MESFL downregulated the mRNA expression of caspase-3 in diabetic rats.

**Conclusion:**

It can be concluded from the data presented in this study that MESFL exerts a protective effect on diabetic rats due to its antidiabetic, antioxidant, antihyperlipidemic and antiapoptotic effects and may be considered a treatment for T2DM.

## Introduction

Diabetes mellitus (DM) is a metabolic condition defined by high blood glucose levels that can be caused by either a lack of or inability to utilize insulin [1, 2]. According to recent estimates by the International Diabetes Federation, a total of 537 million (representing 10.3% of the global adult population) people worldwide are estimated to be living with diabetes, whereas more than 24 million and 7.7 million people in Africa and Nigeria, respectively, are living with diabetes [3]. According to a previous report, more than 2.5% of the world’s population is diagnosed with DM [4]. There is an urgent need to look for viable treatment options given the rising prevalence rate and crippling effects.

Type 2 diabetes (T2D), defined by insulin resistance and partial pancreatic β-cell dysfunction, is the most prevalent type of diabetes and is responsible for more than 90% of diabetic morbidity and mortality [2]. T2D progression has been linked to pancreatic β-cell dysfunction and insulin resistance, resulting in chronic hyperglycemia and dyslipidemia [5–7]. The production of free radicals via diverse pathways, which are triggered by autooxidation of excess glucose in the bloodstream, may account for chronic hyperglycemia [8]. Oxidative stress has been linked to a number of T2D-associated macro- and microvascular complications [9, 10]. Glycated hemoglobin (HbA1c) is an important biomarker for monitoring and managing T2D [11]. When blood glucose binds to hemoglobin, the protein that transports oxygen in red blood cells, HbA1c is generated. Higher blood sugar levels result in higher HbA1c production. HbA1c levels are directly associated with the risk of developing long-term consequences of T2D, such as heart disease, stroke, nerve damage, kidney disease, and eye issues [11, 12].

The pathogenesis of DM is heavily influenced by oxidative stress caused by hyperglycemia, which is characterized by an elevated production of reactive oxygen/nitrogen species (ROS/RNS) and the inhibition of antioxidant defense systems, including SOD, CAT, and GSH [13, 14]. These tissues may be more vulnerable to oxidative stress as a result of an imbalance between ROS/reactive nitrogen species (RNS) and antioxidant defense mechanisms, which could exacerbate DM pathogenesis. Cells are provided with the redox transcription factor Nrf2 to provide cellular protection to counteract oxidative stress [15]. Additionally, it has previously been shown that the Nrf2/Keap1 pathway regulates redox homeostasis predominantly at the transcriptional level [16, 17]. It controls the genes involved in the inflammatory response in addition to playing a crucial upstream regulatory role in the overall antioxidant response [15, 18].

In clinical medicine, evidence that the Keap1/Nrf2 pathway is related to hypoglycemic damage in the liver has attracted increasing amounts of attention [19]. It has been suggested that activating the Nrf2/Keap1 pathway can reverse the oxidative damage caused by diabetes, and clinical research on this antioxidant route has demonstrated significant results. Hence, targeting the Nrf2/Keap1 pathway is a promising therapy for reducing oxidative stress in T2DM patients.

In the present study, the antioxidant, antidiabetic, and antiapoptotic properties of Spilanthes filicaulis were investigated under diabetic conditions. It is a shrubby plant of the family Asteraceae that is often found in tropical and subtropical areas, including India, Malaysia, America, Africa, and West Africa [20]. *Spilanthes filicaulis* spp. is applied in the following ways: snakebite treatment (Ghana) using the aerial part of the plant [21]; emetic treatment (Benin City, Nigeria) using its leaves [22]; intestinal disease and diarrhea treatment (Cameroon); and chest pain, eczema, guinea worm, and toothache treatments (Cameroon) using the whole plant [23]. The entire plant was employed in Babungo, a region in northwestern Cameroon, for various medicinal purposes, such as relieving side pain through enemas, facilitating blood coagulation, applying it topically to the skin as a local anesthetic, treating toothaches and gastritis, and treating malaria [24]. Recently, [25] reported the pharmacological properties of the ethyl acetate fraction of *S*. *filicaulis* leaves. In this work, for the first time, we assessed the efficacy of methanolic extracts of *S*. *filicaulis* leaf extracts as powerful antioxidant, antidiabetic, and antiapoptotic agents. In addition, this study explored the therapeutic potential of the methanolic extract of *Spilanthes filicaulis* leaves on key genes modulating the Nrf2/Keap1, PPAR-γ, and PTP1B pathways, as well as the glucose-metabolizing enzymes in the liver of streptozotocin-induced diabetic rats.

## Materials and methods

### Plant material

*Spilanthes filicaulis* plants were obtained from a local farm settlement in Iwo, Osun State, and the leaves were manually obtained. Authentication and identification of the plant were conducted at the Department of Pure and Applied Biology, Bowen University, Iwo Osun State, with herbarium number BUH035.

### Chemicals

All chemicals and reagents used were of analytical grade, and the Streptozotocin used was procured from Glentham Life Sciences Ltd., Corsham, United Kingdom.

### Experimental animals

Twenty-five healthy male Wistar rats weighing 140–170 g were procured for the purpose of this study. The rats were housed in decontamination cages under specific conditions and acclimatized for one week, with access to rat feed (pellet) and uncontaminated water. In compliance with the criteria and guidelines indicated in the National Institute of Health (NIH) guidelines for the care and usage of laboratory animals, an ethical approval number was provided (BUAC/BCH/2023/0002A).

### Preparation of the *S*. *filicaulis* methanolic extract

The manually obtained leaves were air-dried and mashed. The sample was extracted by immersion of a known mass of leaf powder (34.7 g) in 70% methanol (300 ml) in an airtight beaker, which was left to stand for 72 hours. Filtration of the extract was performed using a muslin cloth, with further concentration using a water bath and oven at 70°C.

#### Animal grouping

The animals were categorized into five groups, with five animals in each category. Group allocation is indicated below.

Group A included the normal control group (nondiabetic mice that received feed and distilled water daily).
Group B included the diabetic control group (untreated, given only feed and water daily).
Group C: Diabetic + Metformin (100 mg/kg body weight)
Group D: Diabetic + *S*. *filicaulis* methanolic extract (low dose of 150 mg/kg).
Group E: Diabetic + *S*. *filicaulis* methanolic extract (high dose of 300 mg/kg).

#### Induction of type 2 diabetes

Diabetes was induced in the diabetic animals in groups B, C, D, and E via the intraperitoneal administration of 40 mg/kg body weight STZ (STZ) prepared in citrate buffer at pH 4.5 after the administration of fructose for two weeks prior to induction. The induction was performed after the animals were fasted for 12 hours; thereafter, the fasting blood glucose level was determined using a glucometer. Blood samples were obtained from the tail end of the rat using a lancet, and animals with fasting blood glucose levels greater than 250 mg/dl after 48 hours were considered diabetic. The experiment was conducted for 14 days [26]. The rat care procedures were approved by the Bowen Animal Ethics Committee, and the study was authorized with the identification number BUAC/BCH/2023/0002A.

#### Animal sacrifice and sample collection

Animal euthanasia was performed by placing the animals under anesthesia with 3 ml of ketamine, which was administered intramuscularly. An incision was made in the abdominal cavity, and the skin was removed to expose the abdominal and thoracic regions. Blood samples were obtained from the right ventricle of the heart using a syringe and left to stand at room temperature undisturbed for approximately 30 minutes before centrifugation at 3000 r.p.m. for 5 minutes. The obtained serum was labeled appropriately for further biochemical analysis. The liver was carefully procured and weighed, after which homogenization was performed using ice cold phosphate buffer in a mortar and pestle. Centrifugation of the homogenates was performed at 5000 rpm for 10 minutes, after which the supernatant was obtained and stored in a refrigerator for further biochemical analysis.

### Determination of biochemical parameters

The standard procedures adopted were as described for fasting blood glucose [27], glycated hemoglobin [28], glutathione-S-transferase (GST) [29], catalase [30], superoxide dismutase (SOD), reduced glutathione (GSH) [31], malondialdehyde (MDA) [32], total cholesterol (TC) and triglycerides [33], low-density lipoprotein-cholesterol (LDL-C), high-density lipoprotein-cholesterol and very low density lipoprotein-cholesterol (VLDL-C) [34], alkaline phosphatase (ALP) [35], aspartate aminotransferase (AST) and alanine aminotransferase (ALT) [36]. Other procedures adopted were as described for glycogen [37], hexokinase (HK) [38], and glucose-6-phosphatase (G6 Pase) [39].

### Gene expression study

#### Isolation of total RNA

Total RNA was extracted from liver samples in cool (4°C) TRIzol reagent using the Quick-RNA MiniPrep^™^ Kit from Zymo Research, USA (Cat: R2050-1-50, Lot: ZRC186885). DNA contamination was eliminated after DNAse I treatment (NEB, Cat: M0303S). The RNA from the supernatant was precipitated using an equivalent amount of isopropanol (Burgoyne Urbidges & Co, India, Cat: 67-63-0). The RNA pellet was washed two times in 70% ethanol (70 ml of absolute ethanol (BDH Analytical Chemicals, Poole, England Cat: 10107-7Y) in 30 ml of nuclease-free water (Inqaba Biotec, West Africa, Lot no: 0596C320, code: E476-500ML)). The pellets were air-dried for 5 min and solubilized in RNA buffer (1 mM sodium citrate, pH 6.4).

#### cDNA conversion

A reverse transcriptase reaction was used to transform one (1) microgram of DNA-free RNA into cDNA with the use of a cDNA synthesis kit using ProtoScript II First-Strand Technology (New England Biolabs). A 2 μl solution containing 100 ng of DNA-free RNA was changed to cDNA by employing the M-MuLV Reverse Transcriptase Kit (NEB, Cat: M0253S) in a 20 μl final volume (2 μl, N^9^ random primer mix; 2 μl, 10X M-MuLV buffer; 1 μl, M-MuLV RT (200 U/μl); 2 μl, 10 mM dNTP; 0.2 μl, RNase Inhibitor (40 U/μl) and 10.8 μl of nuclease-free water). The reaction temperature was set to three different conditions: 65°C for 5 min, 42°C for 1 h, and 80°C for 5 min [27, 40].

#### PCR amplification and agarose gel electrophoresis

Polymerase chain reaction (PCR) was used to amplify the gene of interest using NEB (OneTaqR 2X Master Mix) with the following primers (Inqaba Biotec, Hatfield, South Africa). PCR amplification was performed in a 25 μl reaction mixture containing cDNA, primers (forward and reverse SEE BELOW) and Ready Mix Taq PCR master mix (One Taq Quick-Load 2x, master mix, NEB, Cat: M0486S). The following conditions were used: initial denaturation at 95°C for 5 min, followed by 30 cycles of amplification (denaturation at 95°C for 30 s, annealing for 30 s and extension at 72°C for 60 s) and a final extension at 72°C for 10 min. The amplicons were resolved on a 1.0% agarose gel (Cleaver Scientific Limited: Lot: 14170811) in Tris (RGT reagent, China, Lot: 20170605)-Borate (JHD chemicals, China, Lot 20141117)-EDTA buffer (pH 8.4). The GAPDH gene was used to normalize the relative level of expression of each gene, and quantification of band intensity was performed using ImageJ software (Table 1) [27, 41].

**Table 1:** Primer sequences.

Gene	Forward	Reverse
Nrf2	5’- AGCACATCCAGACAGACACCA-3’	5’- TATCCAGGGCAAGCGACTC-3’
Keap1	5’- AGCAGGCTTTTGGCATCAT-3’	5’- CCGTGTAGGCGAACTCAATTAG-3’
PTP1B	5’-TGTCTGGCTGATACCTGCCTCT-3’	5’-ATCAGCCCCATCCGAAACTTCC-3’
PPAR-γ	5’- TCATGACCAGGGAGTTCCTC-3’	5’- TCAGCGACTGGGACTTTTCT-3’
Caspase-3	5’- GTGGAACTGACGATGATATGGC-3’	5’- CGCAAAGTGACTGGATGAACC-3’
GAPDH	5’-CTGGAGAAACCTGCCAAGTATG-3’	5’- GGTGGAAGAATGGGAGTTGCT-3’

#### Histopathological examination of the liver tissues

A standard laboratory protocol for paraffin embedding was used to treat the formalin-preserved liver and pancreatic tissues of the male rats. Tissue sections (4 mm) were fixed to slides, deparaffinized in *p*-xylene, rehydrated in ethanol (100, 80, 70, and 50%) and rinsed with water. Slides were stained for 5 min in hematoxylin, rinsed with water, counterstained in eosin, mounted in dibutylphthalate polystyrene xylene, cover-slipped, and viewed at 100× with a Leica slide scanner (SCN 4000, Leica Biosystems, Wetzlar, Germany).

### Statistical analysis

The results are expressed as the mean ± SEM (n = 5). The means were analyzed using one-way analysis of variance followed by a Duncan multiple range *post hoc* test (DMRT) at *p* < 0.05 using GraphPad version 8.0 (GraphPad Software, Inc., San Diego, California, USA).

## Results

### Acute oral toxicity profile of *Spilanthes filicaulis*

As shown in Table 2, the methanolic extract of *Spilanthes filicaulis* (MESFL) demonstrated a safe profile in an acute oral toxicity study. MESFL demonstrated a good safety profile, indicating that the plant is nontoxic up to a dose of 1000 mg/kg. Single animal mortality was recorded during the study, as one of the two rats administered a 2000 mg/kg dose died, indicating the safety of MESFL at doses ≤ 1000 mg/kg. Conversely, in rats dosed with 2000 mg/kg, half the sample size died. Thus, the high safety of this plant up to 1000 mg/kg justifies the use of a high dose of 300 mg/kg and a low dose of 150 mg/kg for the treatment.

**Table 2:** Acute oral toxicity profile of the methanolic extract of *Spilanthes filicaulis* leaves on male Wistar rats before oral administration.

S/N	Methanolic extract of *Spilanthes filicaulis* (mg/kg)	Mortality	Adverse effect	One week post treatment observation
1	50	0/3	Itching few hours after administration was observed, this stopped after 16 hours.	No sign of toxicity was observed, and itching stopped completely
2	100	0/3	Initially, itching was observed, this stopped after approximately 16 hours of administration	No sign of toxicity was observed, itching stopped.
3	1000	0/3	Itching was noticed, this stopped after approximately 16 hours after administration	No sign of toxicity was observed, itching stopped.
4	2000	1/3	Death of the first Wistar rat was confirmed after taking 25 ml of the pre calculated 29.4 ml of methanolic leaf extract, 2nd Wistar rat received the complete dosage to account for (2000 mg/kg), mild itching and reduced activity was observed few hours after administration this stops after approximately 16 hours.	Death of the first rat was recorded indicating toxicity, 2nd rat initially suffered from reduced activity indicating toxicity and continuous administration at this dosage will eventually lead to death.

### Effects of MESFL on the biochemical parameters of STZ-induced diabetic rats

#### Fasting blood glucose

**Fig 1:**
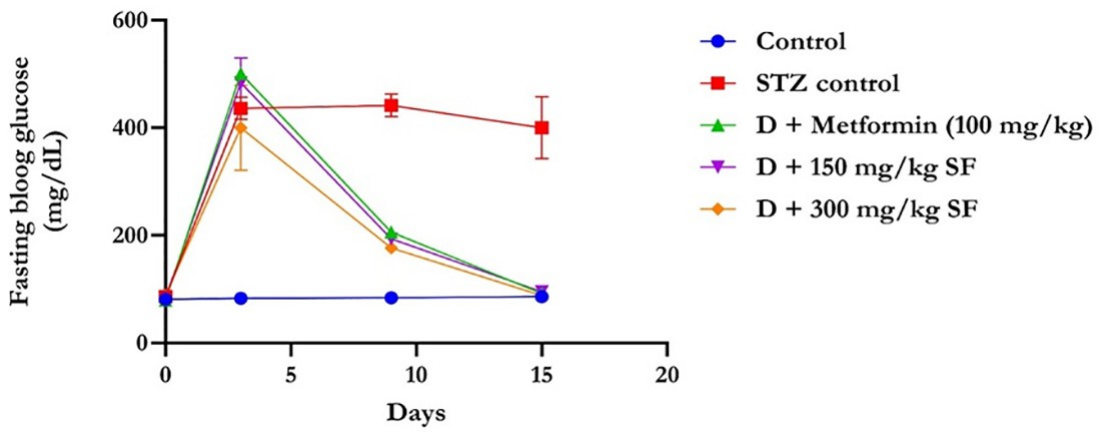
Fasting blood glucose (mg/dl) levels of STZ-induced diabetic rats before and after treatment with the methanolic leaf extract of *Spilanthes filicaulis*. The data are presented as the means ± SDs (n = 5). **STZ**: Streptozotocin **D**: Diabetes, **SF**: *Spilanthes filicaulis*.

Fig 1 shows an observable increase in the fasting blood glucose level of the Wistar rats above 250 mg/dl after 76 hours of STZ induction, and this increase was persistent in the diabetes-untreated group as long as the experiment lasted. The diabetes treatment groups, which were given MESFL at a low dose (150 mg/kg) at 2.4 ml for 160 kg body weight and a high dose (300 mg/kg) at 5.4 ml for 180 kg body weight, as well as the metformin group at a dose of 100 mg/kg, experienced an observable decrease in the fasting blood glucose level. The decrease in blood glucose observed was similar to that in the nondiabetic control group.

#### Body weight and organ body weight

**Fig 2:**
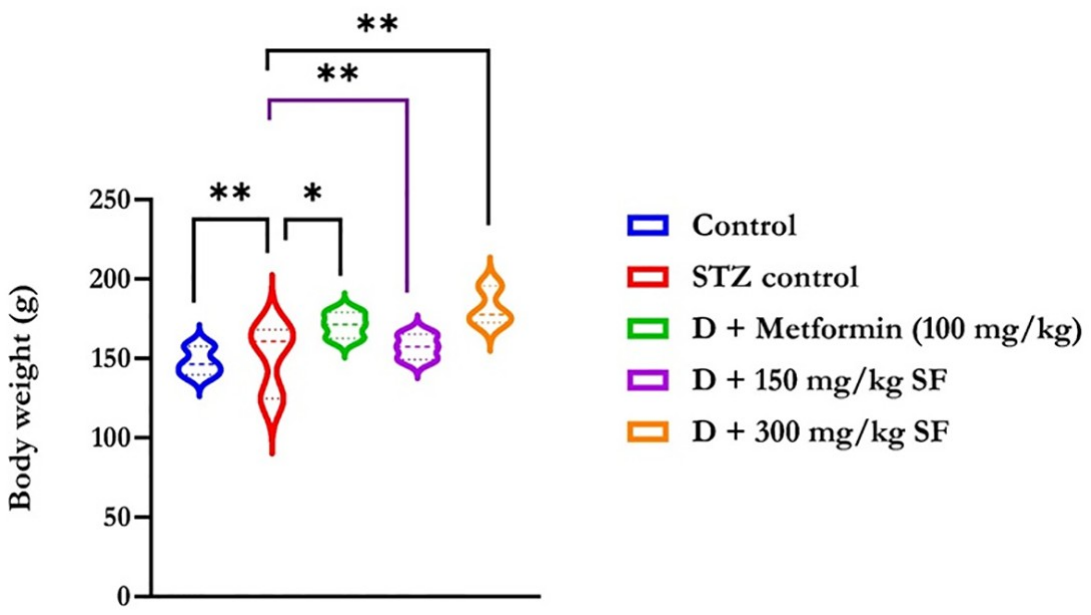
Body weight of STZ-induced Wistar rats before and after oral administration of the methanolic extract of S*pilanthes filicaulis leaves*. The data are presented as the means ± SDs of duplicate determinations. **STZ**: Streptozotocin, **D**: Diabetes.

Fig 2 shows an observable decrease in the weight of the Wistar rats due to the induction of streptozotocin. After administration of the methanolic extract of *Spilanthes filicaulis* leaves at doses of 150 mg/kg and 300 mg/kg, an increase in the body weight of the experimental animals was observed. Conversely, weight loss persisted in the diabetic untreated group throughout the duration of the study. The organ body weight ratios of the liver were similar to those of the Met group.

### Glycated hemoglobin

Administration of fructose and STZ significantly elevated the levels of HbA1c in diabetic rats (Table 3). In contrast, MESFL significantly (*p < 0*.*05*) reduced the levels of glycated hemoglobin compared with the metformin control.

**Table 3:** Glycated hemoglobin levels of streptozotocin-induced Wistar rats after oral administration of a methanolic extract of S*pilanthes filicaulis* leaves.

Groupings	HbA1c
Control	16.18 ± 0.28^a^
Diabetes untreated	53.78 ± 3.17^b^
Diabetic + metformin	30.43 ± 0.90^d^
Diabetic + low dose MESFL (150 mg/kg)	27.89 ± 2.13^c^
Diabetic + high dose MESFL (300 mg/kg)	17.71 ± 1.40^a^

The data are expressed as the means ± SDs (n = 5).

^a-d^Values with different superscripts are significantly different along the column.

**MESFL**: Methanolic extract of *Spilanthes filicaulis* leaves

#### Hepatic antioxidant activities of STZ-induced diabetic rats administered a methanolic extract of *Spilanthes filicaulis* leaves

Table 4 shows an increase in the activities of glutathione-S-transferase (GST), superoxide dismutase (SOD), catalase (CAT), and GSH in the treatment group upon the administration of the methanolic extract of *Spilanthes filicaulis* leaves and metformin, and a decrease in the diabetes-untreated group was observed. A decrease in the MDA content was observed in the rats treated with the methanolic extract of *Spilanthes filicaulis* leaf and metformin, while there was an increase in the MDA content in diabetic untreated rats; malondialdehyde is an oxidative stress marker, and an increase in the MDA content indicates the activity of free radicals.

**Table 4:** Liver antioxidant markers of streptozotocin-induced Wistar rats after oral administration of the methanolic extract of S*pilanthes filicaulis* leaves.

Groupings	SOD (mmol/min/mg protein)	CAT (mmol/min/mg protein)	GSH (μmol/mL)	GST (mmol/min/mg protein)	MDA (μmol/mL)
Control	2.22 ± 0.14^a^	28.77 ± 2.44^a^	93.47 ± 3.03^b^	64.53 ± 1.61^a^	2.04 ± 0.84^b^
Diabetes untreated	1.20 ± 0.04^b^	17.22 ± 0.80^b^	66.54 ± 0.92^a^	20.43 ± 1.91^b^	3.64 ± 0.02^a^
Diabetic + metformin	2.20 ± 0.18^a^	25.88 ± 3.78^a^	89.80 ± 14.06^b^	57.06 ± 1.95^c^	1.15 ± 0.02^b^
Diabetic + low dose MESFL (150 mg/kg)	2.40 ± 0.02^a^	27.75 ± 0.66^a^	95.40 ± 3.49`^b^	56.89 ± 2.44^c^	1.31 ± 0.01^b^
Diabetic + high dose MESFL (300 mg/kg)	2.60 ± 0.02^a^	34.77 ± 0.01^a^^,c^	81.43 ± 5.51^b^	60.75 ± 1.25^a^	1.36 ± 0.01^b^

The data are expressed as the means ± SDs (n = 5).

^a-b^Values with different superscripts are significantly different along the column.

**MESFL**: Methanolic Extract of *Spilanthes filicaulis* leaves.

**MDA**: malondialdehyde, **SOD**: superoxide dismutase, **GSH**: reduced glutathione, **CAT**: catalase

#### Serum lipid profile of streptozotocin-induced diabetic rats treated with the methanolic extract of *Spilanthes filicaulis* leaves

Table 5 shows an increase in total cholesterol (TC), triglyceride (TG), low density lipoprotein (LDL), and very low-density lipoprotein (VLDL) levels in the diabetic untreated rats, along with a reduction in the level of high-density lipoprotein (HDL). Administration of the methanolic extract at a low dose (150 mg/kg) or high dose (300 mg/kg) as well as metformin at a dosage of 100 mg/kg caused a reduction in TC, TG, LDL, and VLDL levels similar to those in the nondiabetic control group, and an increase in HDL was observed in the treatment groups.

**Table 5:** Serum lipid parameters of streptozotocin-induced Wistar rats after oral administration of the methanolic extract of S*pilanthes filicaulis* leaves.

Groupings	TC	TG	HDL	LDL	VLDL
Control	14.13 ± 0.13^a^	7.69 ± 0.11^a^	16.30 ± 0.90^b^	0.62 ± 0.10^b^	1.54 ± 0.10^b^
Diabetes untreated	19.83 ± 1.14^b^	11.25 ± 0.15^b^	12.81 ± 0.75^a^	4.77 ± 0.13^a^	2.25 ± 1.01^a^
Diabetic + metformin	14.87 ± 0.08^a^	7.50 ± 0.28^a^	16.50 ± 0.12^b^	0.13 ± 0.25^b^	1.50 ± 0.16^b^
Diabetic + low dose MESFL (150 mg/kg)	14.83 ± 0.61^a^	7.11 ± 0.14^a^	14.60 ± 0.10^b^	1.19 ± 0.11^b^	1.42 ± 0.28^b^
Diabetic + high dose MESFL (300 mg/kg)	14.48 ± 0.61^a^	7.52 ± 0.35^a^	23.21 ± 4.82^c^	7.23 ± 0.28^b^	1.50 ± 1.74^b^

The data are expressed as the means ± SDs (n = 5).

^a-c^Values with different superscripts are significantly different along the column.

**MESFL**: Methanolic Extract of *Spilanthes filicaulis* leaves.

**TC**: Total Cholesterol **TG**: Triacyl Glyceride **HDL**: High Density Lipoprotein **LDL**: Low Density Lipoprotein **VLDL**: Very Low Density Lipoprotein

#### Carbohydrate-metabolizing enzyme levels in streptozotocin-induced diabetic rats treated with a methanolic extract of *Spilanthes filicaulis* leaves

As shown in Table 6, a significant decrease in the levels of hexokinase and glucose-6-phosphatase was observed in the diabetic group. Conversely, the treatment groups experienced a notable increase after the administration of the methanolic extract of *Spilanthes filicaulis* at a low dose of 150 mg/kg, a high dose of 300 mg/kg and metformin at a dosage of 100 mg/kg.

**Table 6:** Carbohydrate-metabolizing enzyme levels in streptozotocin-induced Wistar rats after oral administration of the methanolic extract of S*pilanthes filicaulis* leaves.

Groupings	Glucose-6-Phosphatase	Hexokinase
Control	48.8 ± 2.23^a^	2.37 ± 0.23^a^
Diabetes untreated	24.2 ± 1.11^b^	0.95 ± 0.02^b^
Diabetic + metformin	44.8 ± 1.10^a^	1.73 ± 0.24^b^
Diabetic + low dose MESFL (150 mg/kg)	44.7 ± 4.82^a^	2.27 ± 3.37^a^
Diabetic + high dose MESFL (300 mg/kg)	55.8 ± 0.38^c^	1.90 ± 0.19^c^

The data are expressed as the means ± SDs (n = 5).

^a-c^Values with different superscripts are significantly different along the column.

**MESFL**: Methanolic extract of *Spilanthes filicaulis* leaves

#### Liver function indices of streptozotocin-induced Wistar rats after oral administration of the methanolic extract of S*pilanthes filicaulis* leaves

Table 7 shows a decrease in the levels of aspartate transaminase (AST), alanine transaminase (ALT), and alkaline phosphatase (ALP) when treated with the methanolic extract of *Spilanthes filicaulis* at a low dose of 150 mg/kg and a high dose of 300 mg/kg as well as metformin at a dosage of 100 mg/kg. The untreated group showed persistent changes in the levels of AST, ALT, and ALP.

**Table 7:** Liver function indices of streptozotocin-induced Wistar rats after oral administration of the methanolic extract of S*pilanthes filicaulis* leaves.

Groupings	AST	ALT	ALP
Control	40.30 ± 4.00^a^	21.70 ± 2.30^a^	14.30 ± 1.60^a^
Diabetes untreated	51.16 ± 1.92^b^	54.00 ± 2.22^b^	28.90 ± 1.20^b^
Diabetic + metformin	40.63 ± 10.01^a^	33.20 ± 5.51^c^	17.30 ± 3.30^a^^,^^c^
Diabetic + low dose MESFL (150 mg/kg)	25.60 ± 2.70^c^	26.23 ± 1.61^a^	9.60 ± 3.10^d^
Diabetic + high dose MESFL (300 mg/kg)	36.83 ± 3.92^a^^,^^d^	32.40 ± 0.94^c^	17.44 ± 0.10^a^^,^^c^

The data are presented as the means ± SDs (n = 5).

^a-d^Values with different superscripts are significantly different along the column.

**MESFL**: Methanolic extract of *Spilanthes filicaulis* leaves

**AST**: Aspartate transaminase; **ALT**: Alanine transaminase; **ALP**: Alkaline phosphatase

### Effects of *Spilanthes filicaulis* methanolic extract on the Keap1/Nrf2, PPAR-γ, and PTP1B signaling pathways

**Fig 3:**
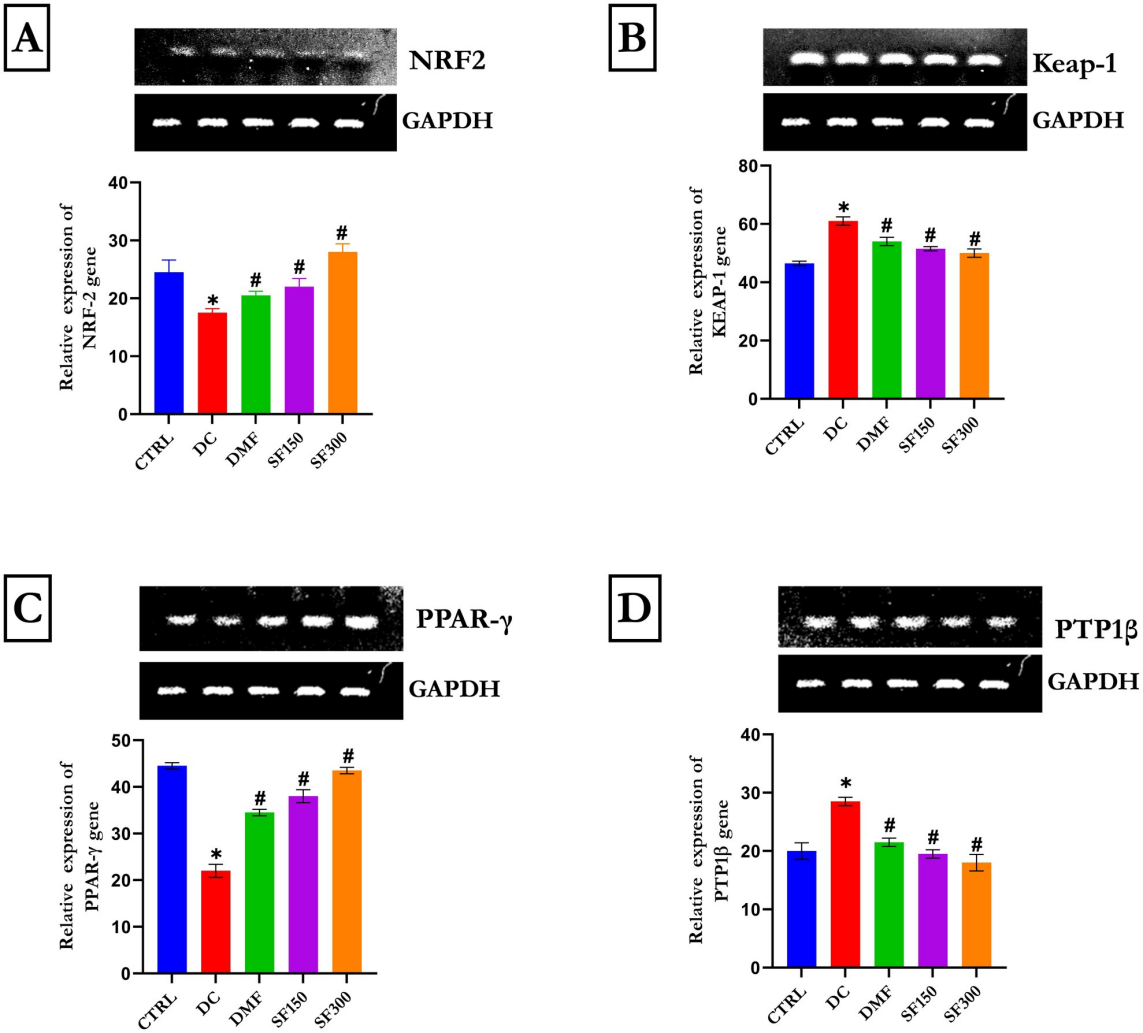
Effects of *Spilanthes filicaulis* on the gene expression levels of (A) Nrf2, (B) Keap 1, (C) PPAR-γ, and (D) PTP1B in the livers of diabetic rats. The data are presented as the means ± SEMs (n = 5). *p < 0.05 compared with the normal control group. #p < 0.05 compared with the diabetic control group. GAPDH was used as a loading protein.

Fig 3A shows that liver Nrf2 expression was lower in diabetic control animals (p < 0.05) than in control rats following the induction of diabetes by STZ. In contrast, a considerable increase in the liver Nrf2 gene was observed in the metformin and MESFL treatment groups relative to the diabetic control group (p < 0.05). Fig 3B shows Keap1, a cellular sensor for the induction of stress. In our study, Keap1 mRNA was significantly overexpressed in diabetic control animals. In contrast, overexpression of Keap1 in the liver of diabetic rats was observed upon administration of metformin and MESFL. Fig 3C reveals the expression of PPAR-γ, which helps maintain glucose homeostasis in the body. In this study, our results showed that PPARγ genes were expressed at lower levels in diabetic control rats (p < 0.05) than in control rats. In contrast, this gene was overexpressed after treatment with metformin and MESFL. Fig 3D shows the gene expression of PTP1B in the livers of diabetic rats. Compared with the diabetic control rats, the control rats had a significantly lower PTP1B gene expression level (p < 0.05). PTP1B gene expression was upregulated in diabetic control rats. In contrast. PTP1B expression was downregulated in the metformin- and MESFL-treated groups (p<0.05) compared with that in the diabetic control group.

### Effect of *Spilanthes filicaulis* methanolic extract on Caspase-3

**Fig 4:**
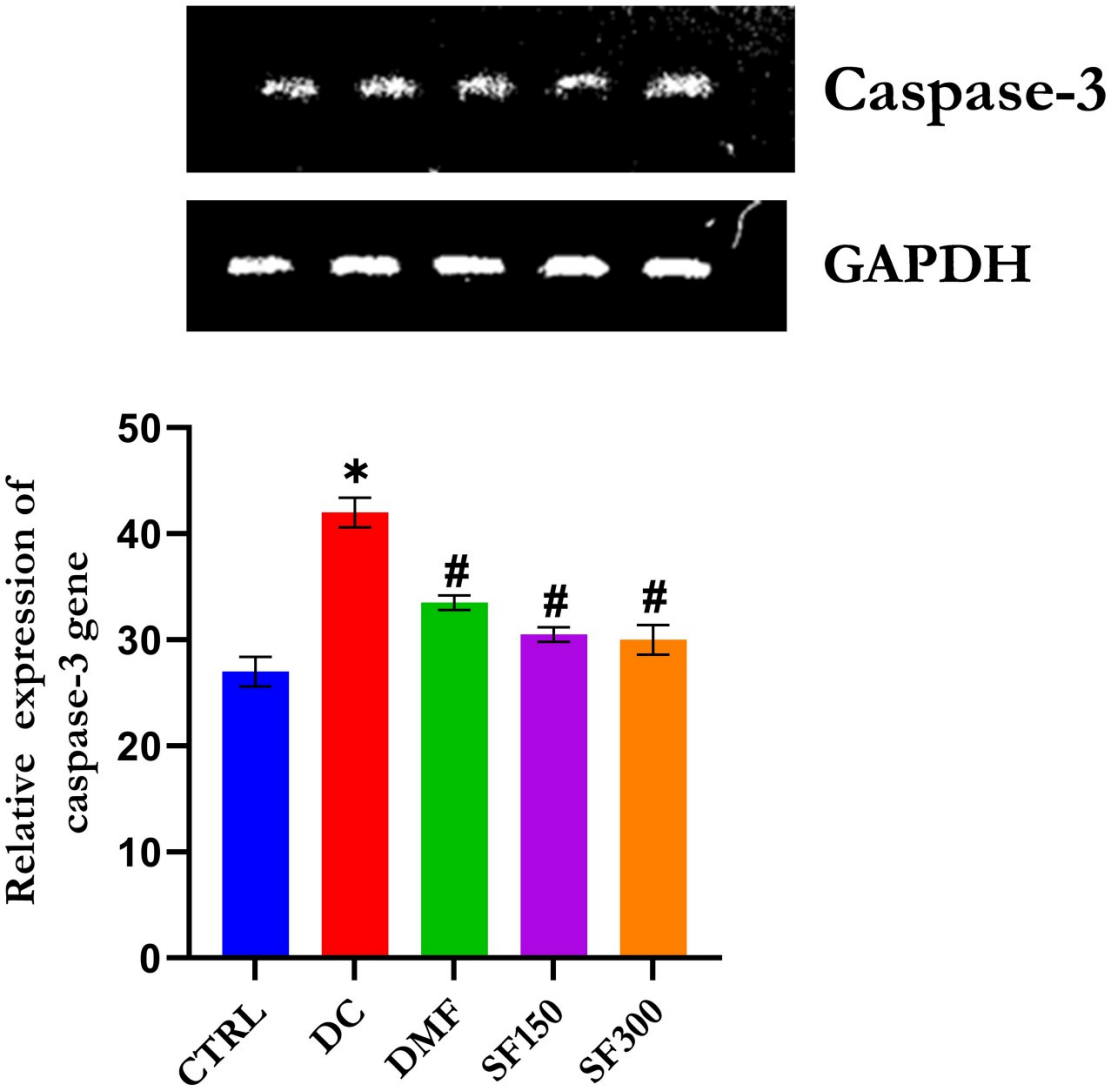
RT‒PCR analysis of caspase 3 in the livers of diabetic rats. The data are presented as the means ± SEMs (n = 5). *p < 0.05 compared with the normal control group. #p < 0.05 compared with the diabetic control group. GAPDH was used as a loading protein.

Fig 4 shows that caspase 3 exhibited significant (p < 0.05) upregulation of caspases in the livers of diabetic rats, which indicated apoptotic cell damage in liver tissues with the progression of DM. Treatment with metformin and MESFL, however, significantly (p < 0.05) arrested the overexpression of caspases in the livers of diabetic rats.

#### Histopathology of the liver tissues

**Fig 5:**
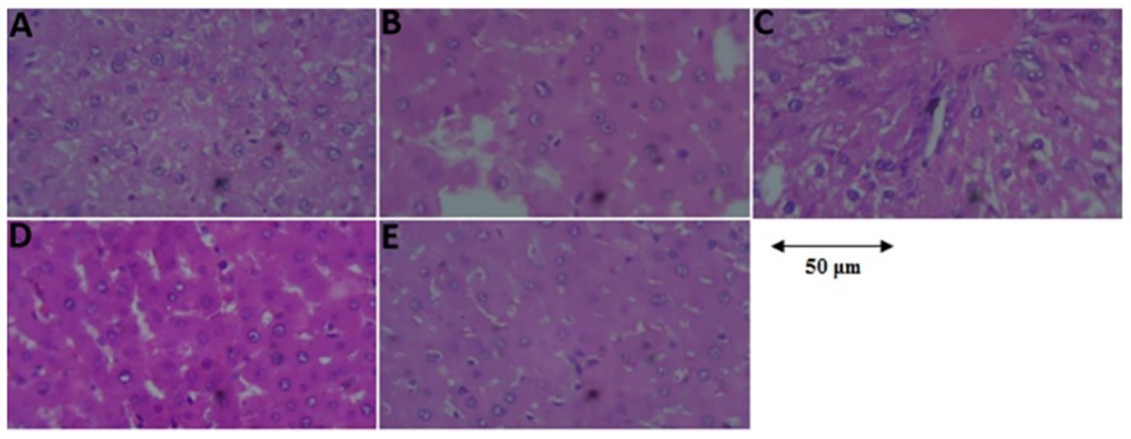
Histological micrographs showing the effect of *Spilanthes filicaulis* and metformin on streptozotocin-induced diabetic rat liver tissues using a hematoxylin and eosin staining histological technique. A (Control), B (Diabetic Control), C (Metformin) D (SF 150 mg/kg), and E (SF 300 mg/kg). **Scale bar**: 50 μm, **magnification**: 400×.

As shown in Fig 5A, there was a normal histoarchitecture of hepatocytes in the control rats, while in the STZ-induced diabetic rats, the liver tissue showed vacuolization, and mild fatty cells were present (Fig 5B). Administration of the methanolic extract of *Spilanthes filicaulis* or metformin resulted in mild vascular congestion as well as intact sinusoids (Fig 5C–5E).

## Discussion

T2DM is a complex health condition that affects almost 90–95% of all patients with diabetes in the general population worldwide [42]. Extracts from plant sources have garnered recognition in recent years for their use in the treatment and management of T2DM [43]. In this study, we administered a combination of fructose and low-dose streptozotocin to experimental rats. This combination led to hyperglycemia with glycemic imbalance, dyslipidemia, and apoptosis. The metabolic characteristics of human T2DM are similar to those of the diabetic rat model. Several researchers have used this experimental model to develop T2DM [43–45].

According to Wilson and Islam [43], fructose- and streptozotocin-induced type 2 diabetes serves as an effective experimental paradigm for examining the antidiabetic effects of various drugs. Fructose and streptozotocin cause T2DM by selectively destroying pancreatic β-cells [43]. Metformin is a widely used drug for diabetes treatment that helps the body process glucose and fatty acids more effectively [46, 47]. We observed an increase in blood glucose levels after 72 hours of fructose and streptozotocin administration. This may be attributed to the selective cytotoxicity of STZ on β-cells [43]. The decrease in serum glucose levels observed in the MESFL-treated and metformin-treated groups compared to the control group supports the idea that the medicinal properties of MESFL may be mediated by secondary metabolites. The decrease in serum glucose levels induced by MESFL may result from an increase in glucose uptake [46, 47], as evidenced by increased glycolysis (increased liver hexokinase activity).

A severe loss in body weight characterizes fructose- and STZ-induced T2DM [48]. Body weight changes as a result of an imbalance between energy intake and expenditure [26, 43]. The reduction in body weight in diabetic rats could be attributed to an alternative energy source provided by enhanced fat metabolism, decreased glucose metabolism, and structural protein degradation [43]. In addition, the increase in body weight observed in diabetic rats following MESFL treatment or metformin therapy may be attributed to the protective effect of *S*. *filicaulis* extract on glucose metabolism by increasing glucose uptake in the liver and reducing the activity of gluconeogenic enzymes, thereby preventing muscle wasting.

Glycated hemoglobin, often measured as HbA1c, has been widely used as an index of glycemic control [49]. Increased nonenzymatic glycosylation is one of the possible mechanisms linking hyperglycemia and vascular complications of diabetes [49]. The increase in HbA1c in diabetic rats could be due to persistent hyperglycemia that stimulates the glycation of hemoglobin [49]. However, the suppression of HbA1c by MESFL could be attributed to its ability, notably at the highest dose, to reduce high blood glucose levels via increased glucose uptake as a consequence of enhanced glycolysis.

Oxidative stress is a condition in which the equilibrium between the production of oxidants and the production of antioxidants is impaired [50]. It is considered to be a common pathophysiology of diabetes-related disorders [51]. The levels of markers of oxidative stress in our study, such as MDA (a byproduct of lipid peroxidation), were significantly elevated, and GST, SOD, and CAT activities and GSH levels were substantially decreased in diabetic rats. Our results are in line with those of previous studies [52]. The administration of 150 or 300 mg/kg MESFL to diabetic rats resulted in significant improvements in the expression of all the tested markers, suggesting that MESFL is an effective antioxidant for the treatment of STZ-induced T2DM.

Streptozotocin-induced T2DM in fructose-fed rats is characterized by lipid abnormalities mostly defined by elevated total cholesterol (TC), triglycerides (TG), LDL, and VLDL as well as a corresponding decrease in HDL [53]. Unlike HDL, which carries cholesterol from the body’s tissues to the liver, where it is broken down and eliminated, a normal level of LDL plays a role in transporting cholesterol from the liver to different parts of the body. Increased levels of LDL cause coronary heart disease by depositing cholesterol in the arteries [54], whereas elevated HDL prevents atherosclerosis by reducing cholesterol deposition [55]. The results of the study revealed that T2DM induced by streptozotocin in rats resulted in high TC, high TG, high LDL, high VLDL, and low HDL. The obtained results agree with other published reports [55–57]. The administration of 150 or 300 MESFL or metformin to diabetic rats significantly alleviated the hyperlipidemic state.

Hexokinase and glucose-6-phosphatase activity in the liver are destabilized in diabetic rats due to hyperglycemia [58]. Streptozotocin is a hepatotoxic drug known to cause damage and disruption of liver function [59]. Hexokinase and glucose-6-phosphatase are key enzymes involved in glucose metabolism [60]. Hexokinase and glucose-6-phosphatase are key enzymes of the glycolytic and gluconeogenic pathways, respectively. The reduced activity of hexokinase in diabetic rats may be caused by reduced glucose metabolism and glucose utilization. Increased activity by MESFL led to improved glucose use for energy production, which indicated increased glycolysis and a greater uptake of glucose from blood by liver cells. Glucose-6-phosphatase activity increases in diabetic rats due to an increase in the synthesis of enzymes that contribute to the increase in glucose production by the liver during diabetes conditions [61]. In addition, MESFL may reduce the activity of glucose-6-phosphatase by inhibiting glycolysis and gluconeogenesis, decreasing gluconeogenesis by increasing insulin secretion, and modulating the activity of this enzyme through the regulation of cyclic adenosine monophosphate (cAMP) or all of the above [47]. These biochemical indicators were significantly restored in diabetic rats treated with MESFL and metformin, thereby maintaining homeostatic regulation of blood glucose levels.

In diabetic rats, liver damage was observed as a result of increased serum ALT, AST and ALP levels. Elevated serum ALT and AST levels are markers of liver damage. Liver membrane integrity is impaired, allowing these enzymes to leak out of the cytosol into the blood stream [62]. These findings are consistent with previous findings of elevated serum AST, ALT, and ALP levels in diabetic rats [63].

Disturbances in intermediary metabolism and hyperglycemia-induced oxidative stress underscore the important etiology of liver injury in diabetes patients [64, 65]. Thus, we explored the antioxidant potential of MESFL in diabetic conditions via modulation of the Keap1/Nrf2 antioxidant pathway. Studies using animal models have demonstrated the critical role of the Keap1/Nrf2 system in the pathogenic defense of cells in diabetic conditions [66, 67]. Low expression of Nrf2 was observed in the livers of diabetic rats, and the findings agree with previously published articles [67–69]. The mechanism underlying the dysregulation of Nrf2 in diabetic patients is unclear; however, therapies that increase Nrf2 mRNA expression may reverse oxidative damage. In contrast, diabetic rats exhibited a significant increase in keap1, a negative regulator, indicating a stronger association between Nrf2 and keap1 and a decrease in Nrf2 translocation into the nucleus to activate antioxidant genes [70]. The results of our study concur with those of several other reports [67–69]. The antioxidant activity of MESFL was demonstrated by increased nuclear translocation of Nrf2 and reduced mRNA expression of Keap1 in the livers of diabetic rats induced by STZ.

Fructose- and STZ-induced diabetic rats exhibited amplified expression of PTP1B. The degree to which certain components and pathways are phosphorylated determines the extent to which insulin signaling is controlled. PTP1B is the most important phosphatase in the insulin signaling cascade and directly phosphorylates insulin receptors (IR and IRS 1) to inhibit them, altering insulin production and increasing blood glucose levels during diabetes [71]. The results of this study indicated a significant decrease in blood glucose levels when MESFL was administered. Additionally, an RT‒PCR-based analysis of diabetic rats treated with MESFL revealed a significant decrease in PTP1B expression. This could have resulted in an increase in glucose-stimulated insulin release, resulting in a significant drop in blood glucose levels [72].

PPARγ plays a critical role in regulating the metabolism of glucose and lipids, both of which are reduced in tissues during diabetes [63]. PPARγ increased significantly in the liver tissue of diabetic rats treated with MESFL or metformin. This finding suggested that PPARγ may play a critical role in the physiopathology of diabetic liver tissues [73].

In the present study, the liver caspase-3 gene was overexpressed in STZ-induced diabetic rats. These findings support previous research demonstrating that STZ significantly promotes apoptosis via caspase-3 activation. The occurrence of apoptosis at the onset of diabetes is thought to be the primary disease-causing factor for DM [74]. Cysteine protease (caspase-3) is one of the key mediators of apoptosis. Caspase-3 has been extensively studied and has been found to be increased in the diabetic state [75]. In our study, we found an association between STZ-induced diabetes and the expression of caspase-3. The liver cells of rats treated with MESFL showed apparent downregulation of caspase-3. Thus, it is possible that the inhibition of caspase-3 is one of the mechanisms by which MESFL protects against diabetic liver injury.

In this study, the histology of the liver was investigated, and light microscopy following the H&E technique revealed that the control rats that received distilled water presented a normal histomorphology and that the hepatocytes appeared intact. In STZ-induced diabetic rats, the liver tissue showed several vacuolizations and mild fatty cells present, but the rats treated with metformin and the methanolic extract of *Spilanthes filicaulis* exhibited an intact sinusoid as well as hepatocytes [76].

## Conclusion

In conclusion, treatment of diabetic rats with MESFL considerably enhanced metabolic changes, dyslipidemia, and oxidative and apoptotic conditions due to its antidiabetic, antihyperlipidemic, antioxidant, and antiapoptotic properties. MESFL effectively reduces oxidative stress, carbohydrate and lipid metabolism, apoptosis, and Keap1/Nrf2, PPAR-γ, and PTP1B expression. These compounds exhibit promising antidiabetic effects on T2DM via their effects on Keap1/Nrf2 and PPAR-γ signaling pathways in the liver and their inhibitory effects on PTP1B and caspase-3 in liver cells. Hence, MESFL could be an ideal treatment option for diabetes mellitus. This study’s limitations include the unavailability of equipment to measure protein expression using western blotting. Additionally, scoring of the histology slides and the 3 HE stained sections of 3 animals of each group in supplementary information could not be achieved, nor could the GSH/GSSG ratio be measured at this time.

## Supporting information

S1 Data (SAV)

S2 Data (SAV)

S1 File (ZIP)

S1 Raw data (ZIP)
