# Supplementary figures and images for: Spilanthes filicaulis (Schumach. & Thonn.) C. D Adam leaf extract prevents assault of streptozotocin on liver cells via inhibition of oxidative stress and activation of the NrF2/Keap1, PPARγ, and PTP1B signaling pathways
Source: PLoS One. 2024 Jun 26;19(6):e0306039. doi: 10.1371/journal.pone.0306039 (PMC11207034; doi:10.1371/journal.pone.0306039)

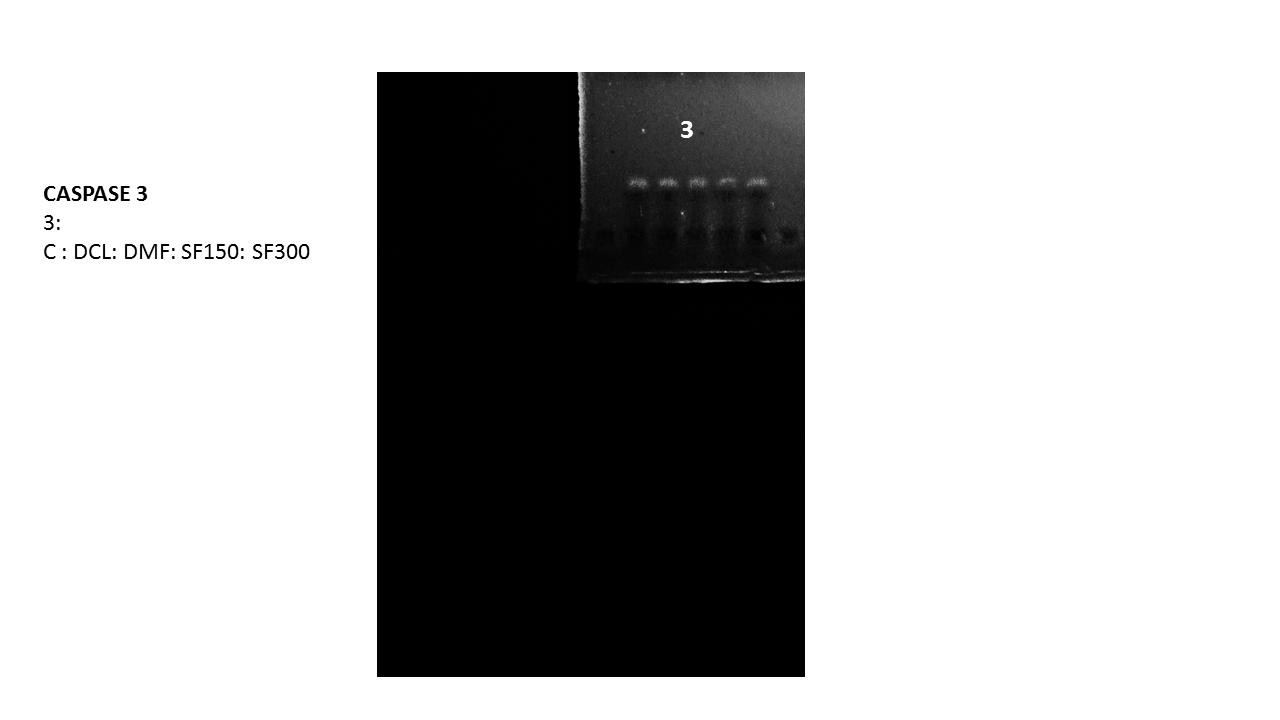

Supplement: S1 File — (ZIP) [file pone.0306039.s003.zip › Caspase 3.TIF]

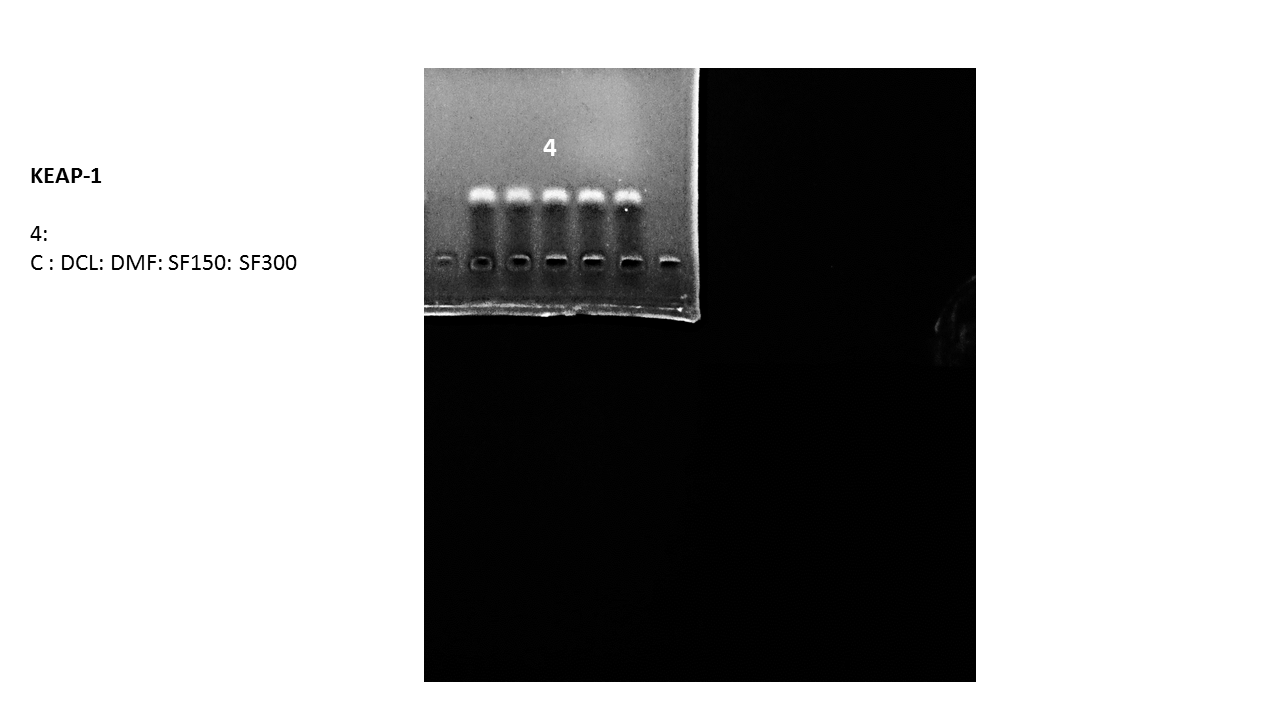

Supplement: S1 File — (ZIP) [file pone.0306039.s003.zip › KEAP1.TIF]

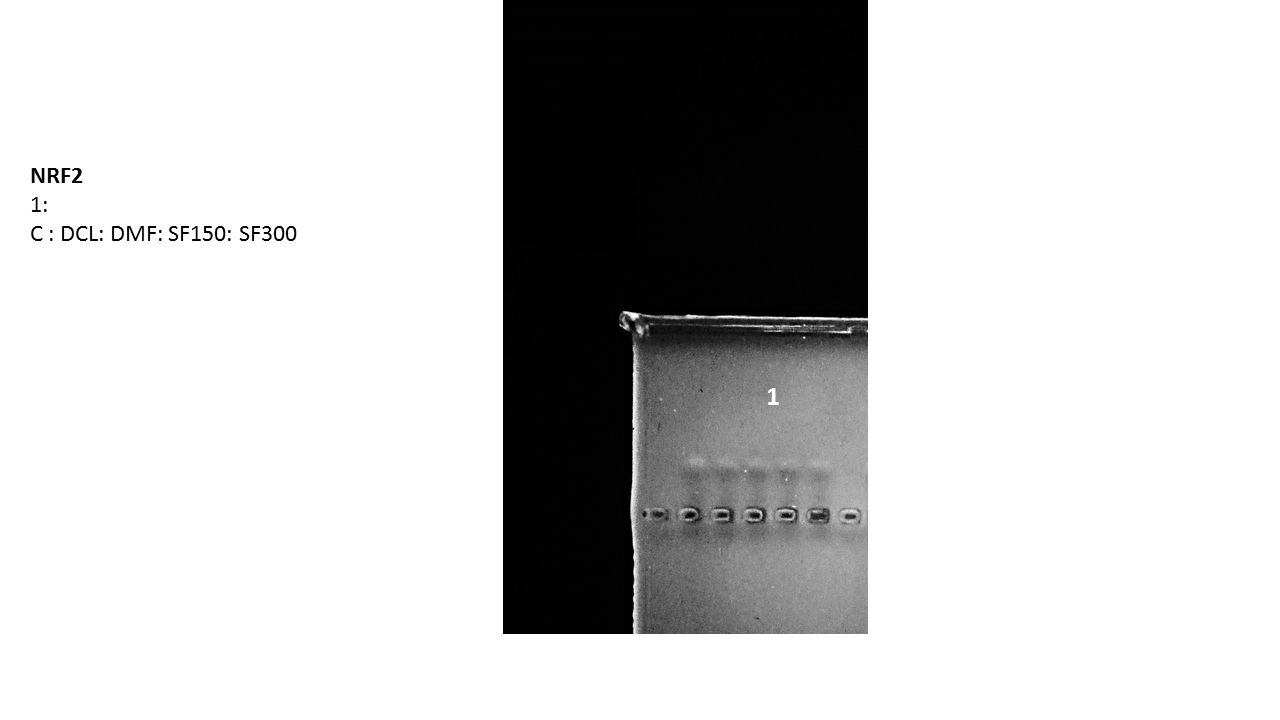

Supplement: S1 File — (ZIP) [file pone.0306039.s003.zip › NRF2.TIF]

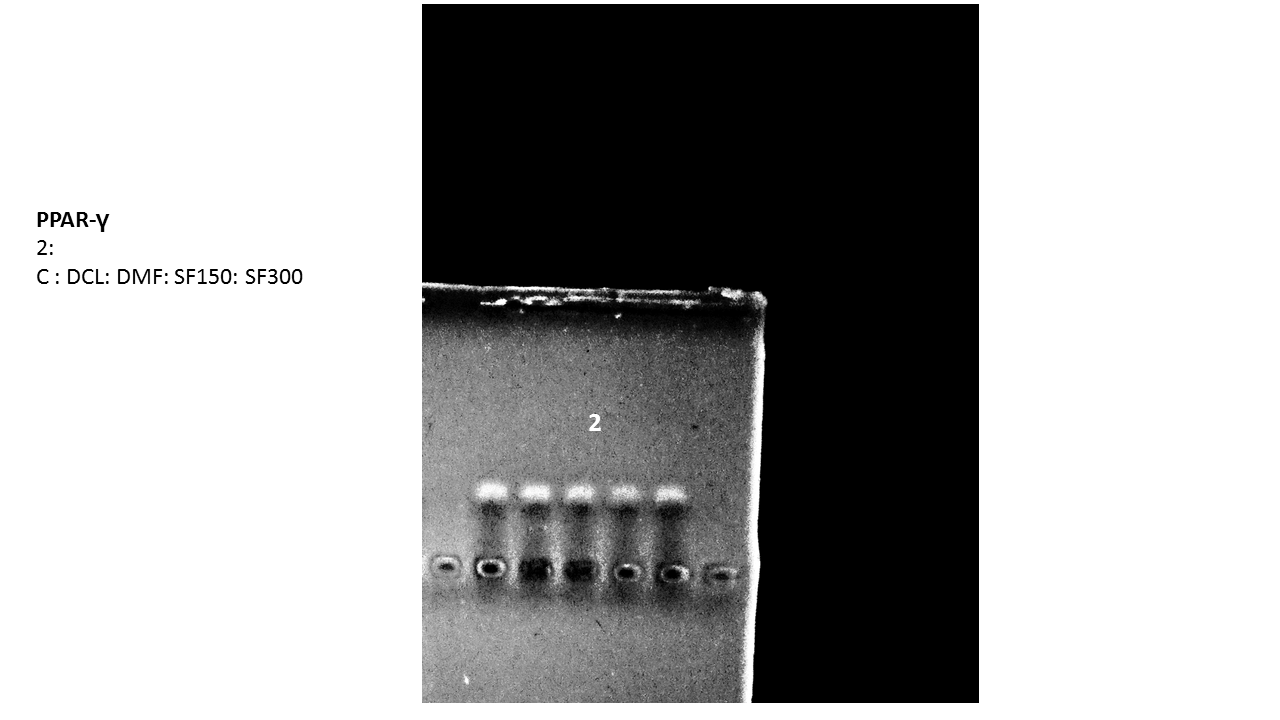

Supplement: S1 File — (ZIP) [file pone.0306039.s003.zip › PPAR-gamma.TIF]

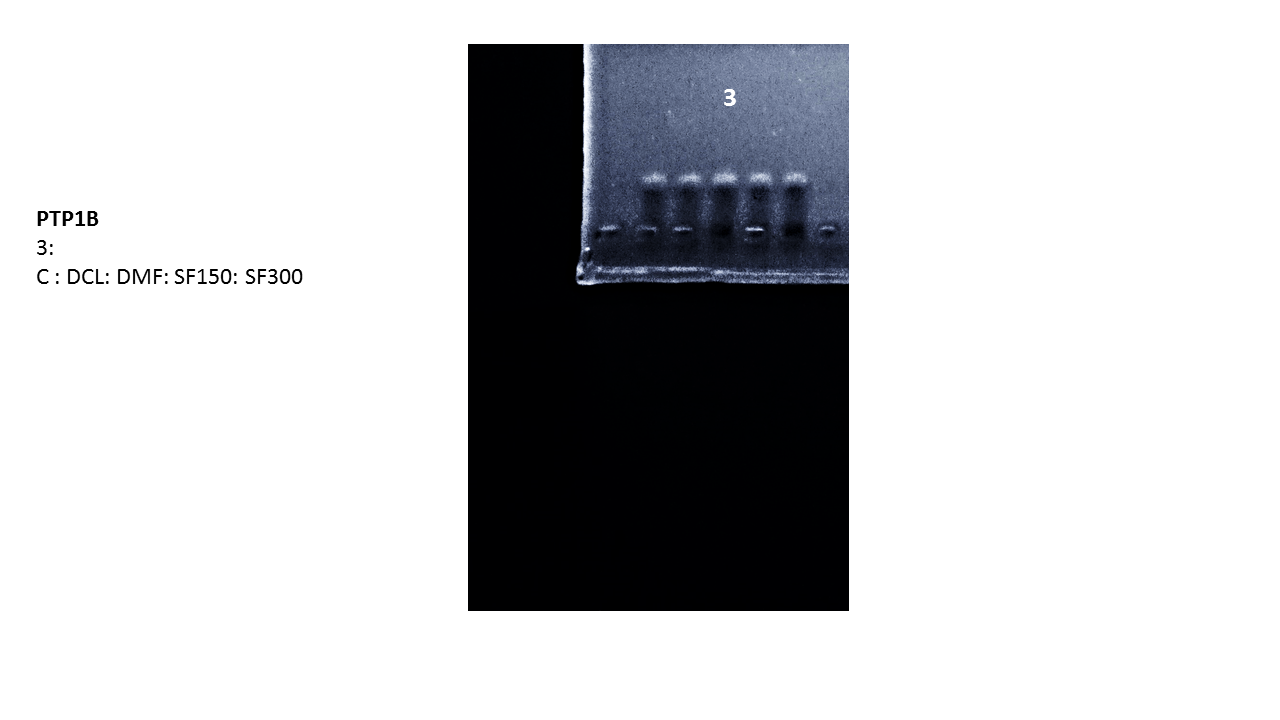

Supplement: S1 File — (ZIP) [file pone.0306039.s003.zip › PTP1B.TIF]
